# Histone Deacetylase 3: A Potential Therapeutic Target for Atherosclerosis

**DOI:** 10.14336/AD.2021.1116

**Published:** 2022-06-01

**Authors:** Li-Ping Jiang, Xiao-Hua Yu, Jin-Zhi Chen, Mi Hu, Yang-Kai Zhang, Hui-Ling Lin, Wan-Ying Tang, Ping-Ping He, Xin-Ping Ouyang

**Affiliations:** ^1^Department of Physiology, Institute of Neuroscience Research, Hengyang Key Laboratory of Neurodegeneration and Cognitive Impairment, Hunan Province Cooperative Innovation Center for Molecular Target New Drug Study, Hengyang Medical School, University of South China, Hunan, China.; ^2^Institute of Clinical Medicine, the Second Affiliated Hospital of Hainan Medical University, Haikou, China.; ^3^School of Nursing, University of South China, Hunan, China

**Keywords:** acetylation, HDAC3, HDAC3 inhibitors, atherosclerosis, cardiovascular diseases

## Abstract

Atherosclerosis, the pathological basis of most cardiovascular disease, is characterized by plaque formation in the intima. Secondary lesions include intraplaque hemorrhage, plaque rupture, and local thrombosis. Vascular endothelial function impairment and smooth muscle cell migration lead to vascular dysfunction, which is conducive to the formation of macrophage-derived foam cells and aggravates inflammatory response and lipid accumulation that cause atherosclerosis. Histone deacetylase (HDAC) is an epigenetic modifying enzyme closely related to chromatin structure and gene transcriptional regulation. Emerging studies have demonstrated that the Class I member HDAC3 of the HDAC super family has cell-specific functions in atherosclerosis, including 1) maintenance of endothelial integrity and functions, 2) regulation of vascular smooth muscle cell proliferation and migration, 3) modulation of macrophage phenotype, and 4) influence on foam cell formation. Although several studies have shown that HDAC3 may be a promising therapeutic target, only a few HDAC3-selective inhibitors have been thoroughly researched and reported. Here, we specifically summarize the impact of HDAC3 and its inhibitors on vascular function, inflammation, lipid accumulation, and plaque stability in the development of atherosclerosis with the hopes of opening up new opportunities for the treatment of cardiovascular diseases.

## 1. Introduction

Atherosclerosis is a lipid-driven chronic inflammatory disease [1] that has become a major contributor to morbidity and mortality worldwide [2, 3]. Through the post-translational modification of the nucleosomal histone, the chromosome regions are converted into transcriptionally active or inactive chromatin [4]. During acetylation modification of histones, histone acetyltransferases (HATs) transfer the acetyl group of acetyl-CoA to the ε-amine of several lysine residues at the amino terminus of histones to enhance gene expression [5, 6]. Conversely, histone deacetylases (HDACs) impede the function of HATs through deacetylation of histone tails, thereby repressing gene expression [6].

The traditional HDAC family and silent information regulator 2 (SIR2) protein family of nicotinamide adenine dinucleotide-dependent Class III HDACs (SIRT1~7) are two subfamilies of 18 proteins with HDAC activity [7, 8]. The classical HDAC family has 11 members and is classified into three groups: class I RPD3-like proteins (HDAC 1, 2, 3, and 8), class II HDA1-like proteins (HDAC 4, 5, 6, 7, 9, and 10), and a solitary class IV HDAC (HDAC11) [4, 6, 9]. HDAC3 is a member of the class I HDACs [10], and its four different splicing variants have been identified as HD3α, -β, -γ, and -δ [11]. HDAC3 is distinctive within the HDAC family because it is expressed not only in the nucleus but also in the cytoplasm and on the plasma membrane [10, 12, 13]. HDAC3 is also a component of the nuclear receptor co-repressor complex and so has distinct molecular and physiological functions [6]. A growing body of evidence shows that HDAC3 has been linked to a variety of disorders, including cardiovascular disease, cancer, neurodegenerative diseases, inflammatory diseases, and metabolic diseases [14]. However, the research done thus far on the role, mechanism, and inhibitors of HDAC3 in cardiovascular diseases is insufficient. This review discusses the role of HDAC3 and its inhibitors in vascular function, inflammation, lipid accumulation and plaque stability associated with the development of atherosclerosis.

## 2. Overview of HDAC3

### 2.1 The structure of HDAC3

The human *HDAC3* gene is found in the synchronization of chromosome 5q31, while mouse *HDAC3* gene is found on chromosome 18B3 [4]. The predicted sequence of HDAC3 is an open reading frame of 428 amino acids with a molecular mass of 49 kDa [4]. HDAC3 is structurally and functionally similar to other Class I HDACs. In particular, the amino acids located between 128 and 196 display a high level of similarity to previously cloned HDAC1 and HDAC2 proteins [15]. Nonetheless, the HDAC3 protein carboxy terminus of amino acids 384 through 428 differs significantly from those of HDAC1 and HDAC2 and occurs in a distinct co-repressor complex with silencing mediators of retinal and thyroid receptors (SMRT) and nuclear receptor corepressors (NCoR) [4, 14, 16-18]. This transcriptional repression regulating multi-subunit complex interacts with numerous genes independent of and distinct from other known HDAC complexes, indicating that HDAC3 may act differently from other HDACs, including HDAC 1 and 2 [4, 19]. The deacetylation activation domain (DAD) contains two SANTs, SANT1 and SANT2, which are located at the N terminus of the SMRT and NCoR. They modulate SMRT activity in NCoR complex during HDAC3 activation [20, 21]. The highly conserved SANT2 region is an important component of the histone interaction domain and is required for the binding and activation of HDAC3 [22, 23].

### 2.2 The functions of HDAC3

HDAC3 removes acetyl from histone and non-histone lysine residues [24]. Lysine acetylation of histone tails is known to neutralize the lysine residues’ positive charge, causing electrostatic interactions with DNA to be reduced while DNA accessibility is increased [25]. Therefore, HDAC3 deacetylation can regulate gene transcription in processes such as cell survival, metabolism, and proliferation through regulating chromatin structure and functions along with certain transcription factors [26, 27]. And HDAC3 represses transcription by deacetylating the histone tail to bind to a specific promoter, so it serves as a repressor for several transcriptional factors, such as c-jun [28] and nuclear factor-κB (NF-κB) [2, 24].

Increasing evidence indicates that a lack of HDAC3 results in isochromatin loss and increases DNA double-chain rupture events, thus affecting cell proliferation [17, 24]. HDAC3 participates in the differentiation of embryonic stem cells to produce endothelial progenitor cells. Thus, it is essential for endothelial cells (ECs) and embryo viability as a survival-promoting biomolecule [29]. For instance, a global loss of HDAC3 causes proliferation defects, which can lead to early embryonic death [29, 30]. Martin [31] et al. demonstrated that HDAC3 homeostasis is critical for the differentiation of stem or progenitor cells into ECs as well as for the endothelial-to-mesenchymal transition and inflammatory response [32].

Conversely, HDAC3 is crucial for the expression of pro-inflammatory gene. In HDAC3-deficient lipopoly-saccharides (LPS)-stimulated macrophages, nearly 50% of the modulating inflammatory gene expression programs failed to activate [2, 33]. HDAC3 has an inhibitory effect on the alternative activation of macrophages stimulated by cytokines *in vivo* and *vitro*, which significantly affects the ability of some organs to respond to inflammatory stimulation [33]. By binding to the macrophage genome at a subset of sites, HDAC3 triggers deacetylation in the tail of family-specific transcription factor-binding histones, some of which cooperate with the transcription factor PU.1 in site-specific and signal-specific macrophage gene expression modulation [33]. Mullican [33] et al. demonstrated that the markers Arg1 and Clec7a, which are involved in alternative activation, undergo induction in HDAC3-deficient macrophages. This suggests that the lack of HDAC3 predisposed macrophages to alternative activation differentiation. Moreover, HDAC3 is a key regulatory factor in macrophage fibrotic phenotype modulation. HDAC3 deletion in macrophages converts them to a phenotype that can increase collagen content and plaque stability [2]. However, this lack of HDAC3 in ECs reduces cell survival and accelerates the development of atherosclerosis [2]. These findings suggest that HDAC3 has a cell-specific function in atherosclerosis.

### 2.3 Expression of HDAC3

**Figure 1.**
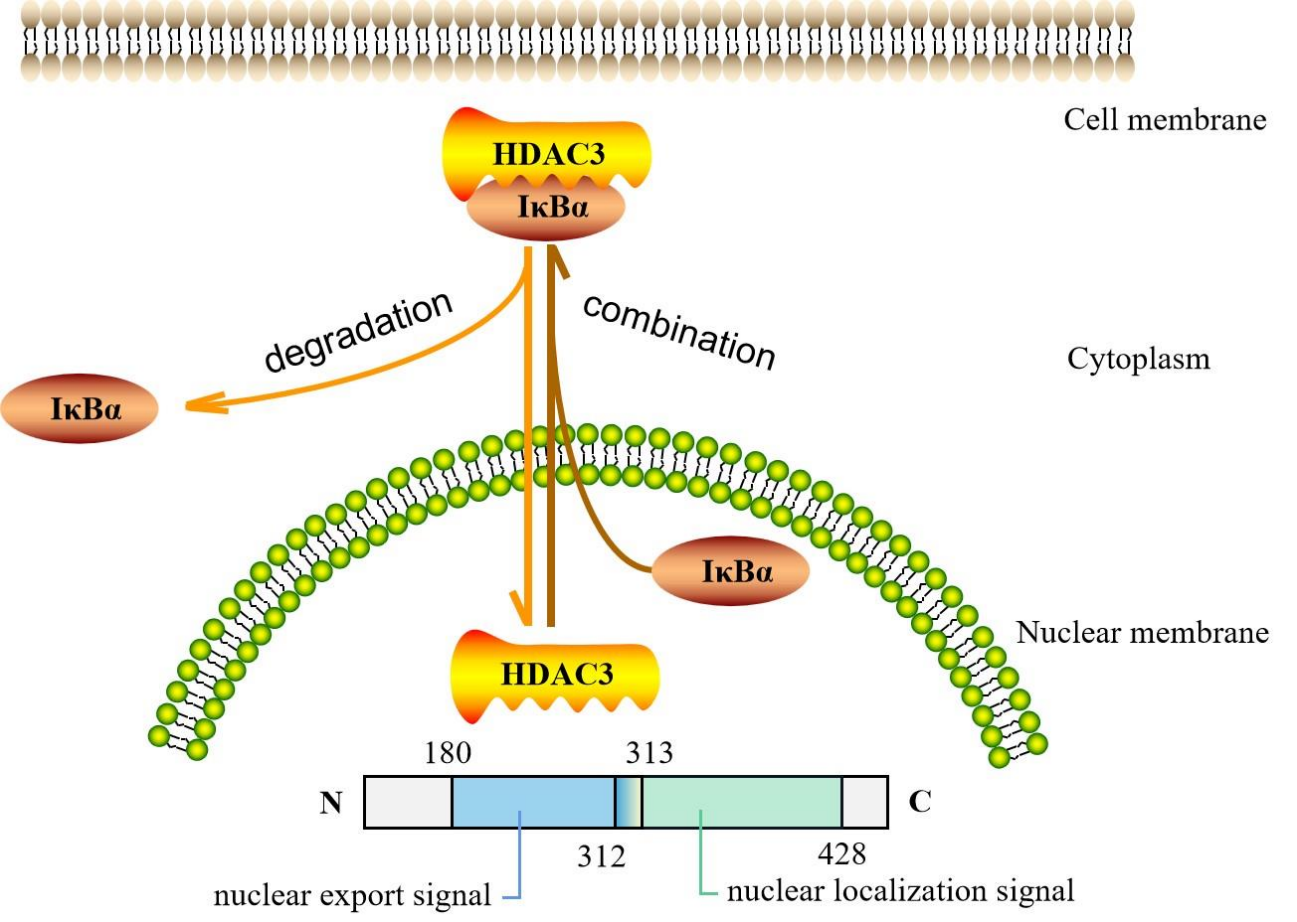
The location of HDAC3 within the cell. HDAC3 may shuttle between the cytoplasm and the nucleus. HDAC3 is maintained in the cytoplasm in combination with inhibitor α of nuclear factor-κB (IκBα) and enters the nucleus when IκBα is degraded. In contrast, when newly synthesized IκBα is present in the nucleus, it binds to nuclear HDAC3 and transfers HDAC3 to the cytoplasm, leading to a redistribution of subcellular HDAC3. Moreover, amino acids 180 through 313 in the central part of HDAC3 act as the nuclear export signal, and amino acids 312 through 428 in the C-terminus act as the nuclear localization signal.

HDAC3 proteins are widely expressed and conserved in many species with low tissue and cell specificity (Table 1). In the liver, HDAC3 most often accompanies essential lipid and genes related to fatty acid metabolism [33, 34]. In fact, HDAC3 in macrophages and liver sarcoplasm rarely overlap, particularly given that its binding is tissue specific [33]. Unlike nuclear-bound HDAC1 and HDAC2 [4], HDAC3 is also present in the cytoplasm and plasma membrane [13] through CRM1-mediated and CRM1-independent pathways [24]. As demonstrated by Li [35] et al., HDAC3 is located in both the cytoplasm and the nucleus [10, 13, 29, 36], suggesting that dynamic localization may modulate the specific function of HDAC3 [13, 37]. Gao [12] et al. reported that HDAC3 may shuttle between the nucleus and cytoplasm, but the factors and mechanisms that trigger this remain unclear. They observed that HDAC3 is maintained in the cytoplasm in combination with inhibitor α of nuclear factor-κB (IκBα) and enters the nucleus when the IκBα is degraded. In contrast, if it is present in the nucleus after IκBα synthesis, it will bind to nuclear HDAC3 and transfer it to the cytoplasm, leading to a redistribution of subcellular HDAC3. Interestingly, Yang [4] et al. found that amino acids 180 through 313 in the central region of HDAC3 act as the signal for nuclear export, while amino acids 312 through 428 in the C-terminus act as the nuclear localization signal (Fig. 1). Thus, the specific nuclear export and nuclear localization structural domains of HDAC3 may be associated with IκBα, although the exact details require further investigation.

**Table 1:** The expression of HDAC3 protein in human tissues and cells.

Organ	Cells	HDAC3 protein expression level
Brain		
Cerebral cortex	Endothelial cells	Medium
	Glial cells	Medium
	Neuronal cells	High
Cerebellum	Cells in granular layer	High
	Cells in molecular layer	High
	Purkinje cells	High
Hippocampus	Glial cells	Medium
	Neuronal cells	Medium
Caudate	Glial cells	Medium
	Neuronal cells	Medium
Endocrine tissues		
Thyroid gland	Glandular cells	Medium
Parathyroid gland	Glandular cells	Low
Adrenal gland	Glandular cells	Medium
Lung		
Nasopharynx	Respiratory epithelial cells	Low
Bronchus	Respiratory epithelial cells	Low
Lung	Alveolar cells	Low
	Macrophages	Low
Proximal digestive tract		
Oral mucosa	Squamous epithelial cells	Medium
Salivary gland	Glandular cells	Low
Esophagus	Squamous epithelial cells	Low
Gastrointestinal tract		
Stomach	Glandular cells	Medium
Duodenum	Glandular cells	Medium
Small intestine	Glandular cells	Medium
Colon	Endothelial cells	Low
	Glandular cells	Medium
	Peripheral nerve/ganglion	Not detected
Rectum	Glandular cells	Medium
Liver	Cholangiocytes	Not detected
	Hepatocytes	Low
Gallbladder	Glandular cells	Medium
Pancreas	Exocrine glandular cells	Low
	Pancreatic endocrine cells	Medium
Kidney	Cells in glomeruli	Medium
	Cells in tubules	Medium
Urinary bladder	Urothelial cells	High
Male tissues		
Epididymis	Glandular cells	High
Seminal vesicle	Glandular cells	Medium
Prostate	Glandular cells	Medium
Female tissues		
Vagina	Squamous epithelial cells	Medium
Ovary	Follicle cells	Low
	Ovarian stroma cells	Low
Fallopian tube	Glandular cells	Medium
Endometrium	Cells in endometrial stroma	Medium
	Glandular cells	Medium
Cervix	Glandular cells	Medium
	Squamous epithelial cells	Medium
Placenta	Decidual cells	Medium
	Trophoblastic cells	Medium
Breast	Adipocytes	Not detected
	Glandular cells	Low
	Myoepithelial cells	Medium
Muscle tissues		
Heart muscle	Cardiomyocytes	Medium
Smooth muscle	Smooth muscle cells	Medium
Skeletal muscle	Myocytes	Low
Soft tissues	Fibroblasts	Medium
	Peripheral nerve	Not detected
Adipose tissue	Adipocytes	Low
Skin	Keratinocytes	Medium
	Fibroblasts	Low
	Melanocytes	Low
lymphoid tissues		
Appendix	Glandular cells	Medium
	Lymphoid tissue	Medium
Spleen	Cells in red pulp	Low
	Cells in white pulp	Low
Lymph node	Germinal center cells	Medium
Tonsil	Germinal center cells	Medium
	Squamous epithelial cells	Medium
Bone marrow	Hematopoietic cells	Low

From the human protein atlas (https://www.proteinatlas.org/)

### 2.4 The regulation of HDAC3

In peripheral blood mononuclear cells, HDAC3 mRNA levels are upregulated by phytohemagglutinin in T cell clones and downregulated by the granulocyte-macrophage colony-stimulating factor [4, 38]. Oleic acid significantly increases HDAC3 mRNA levels, but eicosapentaenoic acid facilitates the proteasomal degradation of HDAC3 proteins, and docosahexaenoic acid suppresses how HDAC3 is expressed in both the post-transcriptional and transcriptional levels in RAW 264.7 macrophages [39]. Accumulating evidence suggests that the expression of HDAC3 is also regulated by miRNAs [40-42]. HDAC3 expression is considerably enhanced in human atherosclerotic lesions and the aortas of apoE^-/-^ mice [2, 29]. In contrast, the inhibition of HDAC3 in bone marrow-derived macrophages found in mice raises the levels of ATP-binding cassette transporter A1 (ABCA1), which is involved in atherosclerosis prevention [43].

Blood flow disturbances also increase the expression of HDAC3 in ECs [29]. According to Zampetaki [29] et al., the treatment of ECs with vascular endothelial growth factor receptor (VEGFR2) and PI3 kinase inhibitors leads to a substantial decrease in HDAC3 protein stability, suggesting that activation of the VEGFR2/PI3 kinase signaling pathway is part of the process that enhances HDAC3 stability. On the other hand, in vitro exposure of ECs to a disturbed flow results in transitory stabilization of the HDAC3 protein and post-transcriptional modification. Similarly, Martin [31] et al. demonstrated that interference flows upregulated both unspliced X-box binding protein 1 (XBP1u) and HDAC3 in a vascular endothelial growth factor receptor and PI3K/AKT-dependent manner. They noted that XBP1 is involved in the upregulation of HDAC3 protein expression [31].

Studies have shown that sulforaphane significantly represses HDAC activity both in vitro, ex vivo and in vivo [44, 45]. More importantly, in a human study, HDAC3 is significantly decreased in the breast biopsy tissue of the group taking the sulforaphane supplement [46]. It has also been discovered that the activity of HDAC in human umbilical vein endothelial cells (HUVECs) is inhibited by short-chain fatty acids (SCFA) [35, 47], especially butyrate [48, 49], propionate and valproic acid [50].

## 3.Role of HDAC3 in the development of atherosclerosis

The location most prone to atherosclerosis is widely recognized as near the branch point of the coronary artery, where disturbed flow readily promotes the appearance and growth of plaque by inducing oxidative stress and inflammation [31]. It is increasingly certain that epigenetics, such as acetylation which alters chromatin accessibility and controls transcriptional responses, regulate several inflammatory and immunological responses that contribute to the development of atherosclerosis [2].

### 3.1 The effect of HDAC3 on vascular function

#### 3.1.1 Regulation of HDAC3 on endothelial cells

The vascular endothelium formed by ECs acts as an osmotic barrier between blood and subendothelial matrix proteins, preventing inflammatory cell infiltration, thrombosis, and proliferation of vascular smooth muscle cells (VSMCs). Endothelial dysfunction is the initial trigger of atherosclerotic lesions and is closely related to dynamic changes in local blood flow [29, 51]. Nitric oxide (NO) is an important mediator for maintaining blood vessel function [52]. Under the action of endothelial nitric oxide synthase (eNOS), L-glycine is used as a substrate to synthesize NO in ECs [53]. However, the reduction of NO levels leads to endothelial dysfunction, increases the permeability of vascular endothelial cells, promotes platelet aggregation, increases leukocyte adhesion, and stimulates cytokine production, thereby contributing to the occurrence and development of atherosclerosis [54].

**Figure 2.**
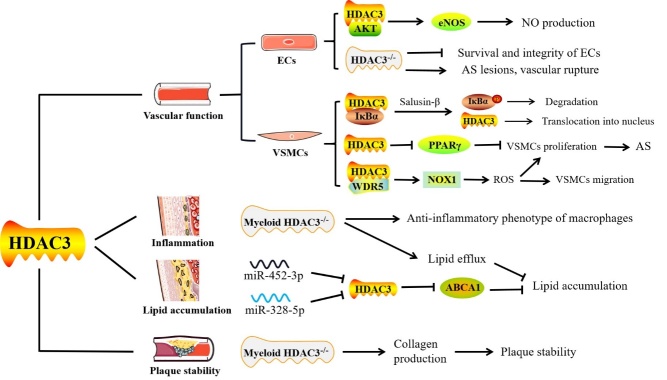
The potential role of HDAC3 in atherosclerosis (AS). In endothelial cells (ECs), the interaction between HDAC3 and AKT is beneficial to the AKT-eNOS signaling pathway and nitric oxide (NO) production. Deletion of HDAC3 in ECs damages cell integrity and survival rates. ApoE^-/-^ mice lacking HDAC3 in the aortas showed severe atherosclerotic lesions and ruptured blood vessels. Furthermore, the IκBα-HDAC3 complex exists in the cytoplasm of vascular smooth muscle cells (VSMCs). Salusin-β treatment induces repression of PPARγ expression due to the nuclear translocation of HDAC3, which may be attributed to the phosphorylation and degradation of IκBα. Moreover, the combination of HDAC3 and WD-40 repeat-containing protein 5 (WDR5) forms a complexe, which positively modulates nicotinamide adenine dinucleotide phosphate oxidase 1 (NOX1), thereby increasing reactive oxygen species (ROS) levels and promoting the transformation of VSMCs into a phenotype with increased cell migration and proliferation. Macrophages in mice lacking myeloid HDAC3 are converted to an anti-inflammatory phenotype and present an enhanced lipid efflux capacity. MiR-452-3p or miR-328-5p inhibits HDAC3 expression by directly targeting 3'UTR but increases the acetylation and expression levels of the ABCA1 gene, thereby reducing lipid accumulation in THP-1 macrophage-derived foam cells. Collagen production by VSMCs is increased in myeloid HDAC3-deficient mice, thereby exhibiting a stable plaque phenotype in the atherosclerotic lesions.

HDAC3 helps to maintain endothelial integrity and function [55]. Interaction between HDAC3 and AKT is beneficial to AKT/eNOS signal transduction and NO production [24, 29]. According to Lee [24] et al., overexpression of programmed cell death 5 (PDCD5) in HUVECs impairs AKT-eNOS signal transduction and NO production by disrupting the interaction of HDAC3 with AKT, ultimately leading to endothelial dysfunction. However, serum NO levels are significantly elevated in mice with endothelial-specific PDCD5 knockout, which helps protect blood vessels from atherosclerosis [24] (Fig. 2). Zampetaki [29] et al. treated aortas isolated from donor apoE^-/-^ mice with shHDAC3 for two hours and then transplanted them into recipient apoE^-/-^ mice. After three weeks, the recipient apoE^-/-^ mice showed severe atherosclerotic lesions, ruptured blood vessels, and broken basement membranes (Fig. 2). Unexpectedly, three of the eight mice who received shHDAC3 allografts perished within two days following surgery. Despite this, it is clear that activation of HDAC3 promotes the differentiation of vascular progenitors into ECs, accelerating reendothelialization of injured arteries and reducing the formation of the neointimum [56].

HDAC3 is also important for the survival of ECs [2]. Zeng [32] et al. demonstrated that shear stress activates HDAC3 via the VEGF receptor 2 (FLK-1)/PI3K/AKT signal pathway, which deacetylates p53 and activates p21. This causes stem cells to develop into ECs and boosts their viability. Consistent with this report, Zampetaki [29] et al. proved that deficiency of HDAC3 in ECs significantly affects cell morphology and leads to a severe cell survival defect, reducing cell numbers by nearly 50%. Results from cell-death assay testing showed that nucleosomes are increased in the cytoplasm of HUVECs after HDAC3 knockout, suggesting the occurrence of cell apoptosis [29]. The knockout of HDAC3 in Tie2-LacZ/apoE^-/-^ mice results in a loss of ECs in the aortic valve segment, indicating that HDAC3 deletion impairs EC survival in *vitro* and *in vivo*[29]. The occurrence of apoptosis induces the cytoplasmic relocation and extracellular release of high-mobility group box 1 (HMGB1), while HDAC3 inhibits the activity of the apoptotic pathway by deacetylating HMGB1 [57]. Suzuki [58] et al. suggested that coupling factor 6 (CF6) contributes to vascular injury and endothelial dysfunction by suppressing the expression of CXC chemokine receptor type 4 (CXCR4), which is a crucial receptor that regulates numerous downstream endothelial effectors. They observed binding of HDAC3 to phosphorylated tyrosine kinase c-Src in HUVECs treated with CF6, which promotes ECs apoptosis and suppresses CXCR4 expression [58]. Altogether, these findings indicate that HDAC3 expression is essential to the maintenance of endothelial survival and function in ECs (Fig. 2).

#### 3.1.2 Regulation of HDAC3 on vascular smooth muscle cells (VSMCs)

After vascular endothelial injury, VSMCs proliferation and migration are the major pathological changes in luminal stenosis and atherosclerotic plaque formation. Due to various local environmental factors, such as increased ox-LDL, human aortic smooth muscle cells (HASMCs) may be transformed into a synthetic phenotype characterized by increased cell migration and proliferation [59]. Zhang [59] et al. demonstrated that the combination of WD-40 repeat-containing protein 5 (WDR5) and HDAC3 forms a complex, which positively modulates the expression of target genes including nicotinamide adenine dinucleotide phosphate oxidase 1 (NOX1) through histone modification. This in turn increases reactive oxygen species (ROS) levels and promotes the transformation of HASMCs into a phenotype with increased cell migration and proliferation (Fig. 2) [60]. Furthermore, Yan [61] et al. demonstrated that cyclic strain of 1 Hz at 10% elongation for 48 hours substantially decreased VSMCs migration compared to static strain. Acetylated histone H3 expression was substantially higher in stressed VSMCs than in quiescent ones. It should be noted that the expression of HDAC3 and HDAC4 was dramatically downregulated by strain, while HDAC7 expression was up-regulated. Changes in the expression of HDAC 3, 4 and 7 may be partly liable for the hyperacetylation of histone H3. However, the specific mechanism and exact role of HDAC3 in the migration of VSMCs still needs to be explored by more in-depth studies.

VSMCs are also involved in the formation of foam cells and development of atherosclerosis [62]. Several studies have shown that disrupting peroxisome proliferator-activated receptor (PPAR) activity in VSMCs results in vascular dysfunction and atherosclerosis exacerbation [63-66]. Many preclinical and clinical research studies have shown that PPARγ agonists reduce atherosclerosis and intimal hyperplasia [64, 67, 68]. Salusin-β is a putative pro-atherogenic factor that adversely regulates the expression of PPARγ in VSMCs, resulting in VSMC proliferation and inflammation *in vitro*[66]. Mechanistically, the IκBα/HDAC3 complex is present in the cytoplasm of VSMCs. However, despite this, treatment with salusin-β induces repression of PPARγ expression due to the nuclear translocation of HDAC3, which may be attributed to the phosphorylation and degradation of IκBα [66]. Similarly, Subramanian [69] et al. demonstrated that PPARγ ligands, thiazolidinedione compounds, inhibit angiotensin II (Ang II)-induced atherosclerosis by interacting with VSMCs-specific PPARγ in hypercholesterolemia mice. Mechanistically, AngII enhanced HDAC3-mediated inhibition of PPARγ through the activation of transforming growth factor β (TGF-β)-p38 mitogen-activated protein kinase in VSMCs [70] (Fig. 2).

### 3.2 HDAC3 is involved in the inflammatory response to atherosclerosis

Excessive pro-inflammatory cytokine production, including IL-1β, TNF-α, and IFN-γ [50], down-regulates ABCA1 expression and promotes the conversion of macrophages to foam cells. Activated macrophages release pro-inflammatory cytokines, which in turn exacerbate the inflammatory response and promote the development of atherosclerosis [71]. As a result, treatment targeting pro-inflammatory cytokines is a promising technique for the treatment of atherosclerosis. HDACs not only play an important role in the innate immune response [2, 72], but also control the expression of inflammatory genes, as evidenced by the potent anti-inflammatory properties of pan-HDAC inhibitors [25].

Macrophages in mice lacking myeloid HDAC3 are converted to an anti-inflammatory phenotype ^[2]^ (Figure 2). Th2 cytokines, like IL-4, stimulate the differentiation of macrophages into alternative activation, which is controlled by a transcriptional pathway affected by epigenomic changes such as histone acetylation [33, 73]. Notably, macrophages deficient in HDAC3 have a polarized behavior similar to IL-4-induced alternative activation. Thus, they generate less inflammatory cytokines and metabolites, thereby lowering inflammation and promoting wound healing [25, 33, 74]. Mullican [33] et al. demonstrated that most of the genes elevated in HDAC3 knockout macrophages are genes that IL-4 positively controls in wild-type macrophages. At the same time, the genes reduced are genes that IL-4 negatively regulates in wild-type macrophages. This trend suggests that the gene expression program induced by HDAC3 deletion in macrophages is strikingly similar to the gene expression program associated with alternative activation. In line with this, Chen [25] et al. discovered that even when stimulated by LPS, HDAC3-deficient macrophages fail to activate over half of their inflammatory gene expression programs.

HDAC3-deficient cells may activate antioxidant responses due to the need to cope with oxidative stress, as the most upregulated genes in HDAC3-deficient macrophages are Nrf2-dependent antioxidant genes [33]. In the entire genome of macrophages, HDAC3 deacetylated histone tails only in the regulatory region, resulting in the suppression of many IL-4 regulated genes with alternative activation characteristics [75]. Thus, HDAC3 acts as a “brake” in the alternative activation of macrophages, and its release may contribute to the treatment of various inflammatory diseases [33]. However, the existing pan-HDAC inhibitors did not show the same effect on macrophage polarization as the deletion of HDAC3. This result may be due to the loss of HDAC3-mediated nuclear receptor suppression, which in turn results in the acetylation of thousands of gene locus and related gene de-inhibition. Meanwhile, pan-HDAC inhibitors have the effect of inhibiting a variety of different HDACs and off-target effects [33].

HDAC3 has an important role in regulating inflammatory gene expression [76, 77] and the migration of monocytes to inflammatory sites [18]. Chen [78] et al. found that overexpression of HDAC3 increased the expression of IL-6, ICAM-1 and MCP-1, but inhibition or knockdown of HDAC3 lowered the expression of vascular cell adhesion molecule-1 (VCAM-1) [79], thus limiting monocyte adhesion [80]. Likewise, Zhu [81] et al. also suggested that HDAC3 has crucial role in regulating TNF-α production. They revealed that LPS stimulates HDAC3 activity via mitochondria reactive oxygen species and c-Src signaling, thereby promoting the expression of TNF-α in cardiomyocytes [81]. Conversely, Lkhagva [55] et al. reported that TNF-α increased the activities and expressions of Class I HDAC (HDAC 1, 2, 3, and 8) in HL-1 cells, while Mahlknecht [82] et al. reported that HDAC3 inhibits the upregulation of TNF-α mRNA induced by LPS in mononuclear phagocytes. One explanation for this contradiction is that the regulation of TNF-α by HDAC3 may be cell-specific [81].

Furthermore, TNF-α regulation of PPARγ activity has been linked to the etiology of atherosclerosis [83]. Jiang [84] et al. suggested that the acetylation modification of PPARγ induces its function and is a ligand-independent activation method. Importantly, they observed that the interaction of HDAC3 and PPARγ deacetylated the PPARγ protein and that acetylation caused by the inhibition of HDAC3 is sufficient to induce PPARγ activity at the transcriptional level. Wang [85] et al. found that PPARγ and HDAC3 were minimally expressed in ox-LDL-induced HUVECs and atherosclerotic mice arterial tissues, but NF-κB/p65 and miR-19b were strongly expressed. Remarkably, overexpression of HDAC3 decreased miR-19b expression and dramatically lowered TNF-α and IL-1β levels in the serum of HFD-fed apoE^-/-^ mice. Mechanistically, PPARγ was targeted and negatively regulated by miR-19b and degraded NF-κB/p65 through ubiquitination, thereby suppressing the inflammatory response induced by ox-LDL in HUVECs [85]. However, miR-19b may target and modulate other genes in addition to PPARγ, and these interactions may also play a role in advancing atherosclerosis. Still, whether HDAC3 intervention in animals or humans has fewer adverse effects and stronger anti-atherosclerotic benefits than miR-19b intervention warrants additional exploration.

In a study that further explored of the relationship between PPARγ and HDAC3, biopsies of subcutaneous adipose tissue in human subjects showed that although mRNA levels of either NCoR or HDAC3 are not correlated with body massindex and insulin sensitivity, they are both positively associated with PPARγ2 mRNA levels [86]. Finlin [86] et al. suggested that because the synergistic regulation of PPARγ1/2, HDAC3 and NCoR in human macrophages may be stricter than in adipocytes, the regulation of PPARs activity by HDAC3 and NCoR may play a crucial role in the negative regulation of pyoglitazone on macrophage-mediated inflammation.

### 3.3 HDAC3 is involved in the lipid accumulation in atherosclerosis

Atherosclerosis is defined by excessive lipid accumulation in the intima of arteries [40, 87, 88]. During atherosclerosis, circulating monocytes are transferred to the subintima where they later differentiate into macrophages. They take up vast quantities of oxidized low-density lipid protein (ox-LDL) to form the foam cells, which are characteristic of early atherosclerotic lesions [88]. According to Zhong [89] et al., the release of acetaldehyde dehydrogenase 2 into the nucleus suppresses transcription of the lysosomal proton pump protein ATP6V0E2 by interacting with HDAC3. Thus, ultimately leads to increased foam cell development. Notably, ATP6V0E2 is one of the key proteins that are responsible for maintaining ox-LDL degradation, autophagy, and lysosomal function [89].

In lesions from HDAC3^fl/fl^-LysMCre (HDAC3^del^) transplanted mice, the lipid content of the plaque and the size of the macrophages decreased, indicating a reduction in foam cells formation. Consistently, both the PPARγ/LXR pathway and cholesterol efflux were up-regulated in HDAC3^del^ macrophages [2] (Figure 2). Our previous study showed that HDAC3 expression was significantly increased in the aortas and mouse peritoneal macrophages (MPMs) of apoE^-/-^ mice overexpressing lncRNA kcnq1 overlapping transcript 1 (kcnq1ot1). As HDAC3 is a direct target of miR-452-3p, whereas Kcnq1ot1 is a competitive endogenous RNA of miR-452-3p which raised HDAC3 expression, resulting in reduced ABCA1 expression and cholesterol efflux, as well as enhanced foam cell formation [88]. ^.^On the other hand, Huang [40] et al. demonstrated that overexpression of miR-328-5p in THP-1-derived macrophages inhibits HDAC3 expression while increasing acetylation and expression levels of the ABCA1 gene. MiR-328-5p inhibits HDAC3 expression by directly targeting 3'UTR, boosting ABCA1 expression and consequent cholesterol excretion while decreasing lipid buildup in THP-1 macrophage-derived foam cells. In line with this finding, Huang [90] et al. demonstrated that dexamethasone increases the protein-protein interaction between the glucocorticoid receptor and HDAC3, lowering the expression levels of ABCA1, ABCG1, and LXRα in the placental trophoblast, which results in disrupted placental cholesterol transport.

### 3.4 Effect of HDAC3 on atherosclerotic plaque stability

Lipid-laden foam cells typically make up the majority of macrophages seen in atherosclerotic plaques and are associated with the main features of plaque instability [2]. Vascular dysfunction, inflammation, and lipid accumulation dominate the occurrence and progression of atherosclerosis, while expanded necrotic core and thinned fibrous cap damage plaque stability, leading to adverse cardiovascular events [3].

HDAC3 was the only HDAC shown to be elevated in ruptured human atherosclerotic lesions, and its expression was found to be negatively associated with pro-fibrotic TGF-β expression [2]. Hoeksema [2] et al. detailed a two-fold thickening of the fibrous cap and an increase in the most mature and stable red collagen subtype, which was observed in lesions from myeloid HDAC3-deficient mice that exhibited a stable plaque phenotype. Immunohistochemical analysis of the plaques displayed that although there was no difference in the total macrophage area between groups, the absence of myeloid HDAC3 resulted in an increase in Dectin1^+^ macrophages in the lesions. Dectin1, a marker of alternative macrophage activation, was found to be especially upregulated in wound healing, tissue repair, and fibrosis, in which TGF-β is a key mediator and frequently acts as an anti-atherosclerotic cytokine that suppresses the immune response and stabilizes atherosclerotic lesions [91]. HDAC3 directly attaches to the TGF-β1 promoter to deacetylate it according to its mechanism. In line with this, Hoeksema [2] et al. observed an increase in histone acetylation at the TGF-β1 locus in ox-LDL-stimulated HDAC3^del^ macrophages.

Similarly, HDAC3^del^ peritoneal macrophages from transplanted LDLR^-/-^ mice demonstrated elevated TGF-β1 expression, as well as histone acetylation, in the same area. Thus, HDAC3 targeted the TGF-locus, and its deletion resulted in hyperacetylation. This in turn enhanced TGF-β1 expression and release, increasing collagen synthesis by VSMCs (Fig. 2). These findings point to the idea that macrophages perform a specialized role in atherosclerotic plaques' fibrosis and identify TGF-β as a critical pro-fibrotic phenotype modulator of myeloid HDAC3-deficient macrophages.

## 4. Applications of HDAC3 inhibitors

Although broad-spectrum inhibitors of histone deacetylases (HDACi) [92] are available for treatment [93], HDACs regulate the acetylation of lysine in thousands of proteins, such as transcriptional regulators [94]. Therefore, gene suppression caused by broad-spectrum HDACi inevitably leads to secondary transcriptional effects, resulting in potential toxicity and side effects [25]. For instance, Choi [95] et al. administered trichostatin intraperitoneally to LDLR^-/-^ mice fed an atherogenic diet once every other day for four weeks and observed aggravated atherosclerotic lesions but no significant changes in blood lipids. Broad-spectrum HDAC inhibition has both pro-inflammatory [2, 25] and anti-inflammatory [96, 97] effects. HDACi is also pleiotropic, lacks specificity for particular HDACs, and has unique off-target effects. Bossche [43] et al. used selective HDAC inhibitors and discovered that suppression of HDAC3 had the atherogenic protective effect of pan-HDAC inhibitors [43]. HDAC inhibition in macrophages, particularly HDAC3, demonstrated anti-atherogenic effects because it partially decreased M1 activation without increasing foam cells [43]. Another HDAC inhibitor, MS-275 [98], was found to effectively inhibit the activities of HDAC1, 2, and 3 [5]. Kashio [99] et al. reported that MS-275 repressed HDAC3 activity in ECs, thereby suppressing the expression of roundabout guidance receptor 4 (Robo4) and inducing endothelial hyperpermeability and extravasation.

The development of small molecule inhibitors for targeted and effective HDAC3 is actively underway [100]. BRD3308 [101], a highly selective HDAC3 inhibitor, has been shown in vitro to reduce pancreatic β-cell apoptosis triggered by inflammatory cytokines or glucolipotoxic stress while increasing insulin production. RGFP-966, another HDAC3-selective inhibitor with an IC50 of 80 nM, had no impact on other HDACs at dosages up to 15 μM [49]. Wu [102] et al. illustrated that RGFP-966 promoted the synthesis and release of fibroblast growth factor 21 (FGF21), which prevented aortic damage caused by diabetes. Increased plasma FGF21 levels might be attributed to the fact that HDAC3 inhibition can prevent diabetes-induced liver damage and enhance FGF21 expression and circulating secretion, including in diabetic conditions [103]. Although HDAC3-specific inhibitors coupled with Cox-1/2 inhibitors resulted in down-regulation of only HDAC3-dependent genes, like certain inflammatory factors, Cox-1/2 inhibitors could also be employed as a partial antidote to minimize the potentially harmful effects of HDAC3 inhibitors in some individuals [25]. Chen et al. [78] discovered that HDAC3 expression was up-regulated and endothelial-to-mesenchymal transition (EndMT) occurred more clearly in the aortas of apoE^-/-^ mice in comparison to the C57BL/6J mice, whereas RGFP966 reduced atherosclerotic lesions and blocked EndMT of the atherosclerotic plaque. Zhao et al. [104] demonstrated that inhibiting HDAC3 with RGFP966 activated PPARγ by increasing its protein acetylation, thus guarding against a reoxygenation-induced increase in transendothelial cell permeability and oxygen-glucose deprivation. These findings highlight an urgent need to develop effective small-molecule HDAC3 inhibitors or target downstream pathways to increase the applicability of therapy for atherosclerotic cardiovascular disease, when overall inhibition of HDACs not only fails to provide additional benefits but may also increase side effects.

## 5. Conclusion and perspective

As an enzyme family crucial to gene transcription and chromatin remodeling, HDACs have been identified as a potentially effective target for treating a variety of diseases [22, 105]. As described in this review, many studies have explored the effects of HDAC3 in vascular function, inflammatory response, lipid accumulation, and atherosclerotic plaque stability, all of which are implicated in the occurrence and progression of atherosclerosis. Pharmaceutical companies are currently looking for chromatin-modifying enzyme inhibitors to treat inflammatory diseases, which is also valuable for the treatment of atherosclerosis, as the histone acetylation of lysine residues by histone acetyltransferase is related to transcription activation and is offset by HDACs. Cumulative researches on the subject strongly indicate that the prevention of HDAC3 in macrophages is an attractive new therapeutic target in cardiovascular diseases [43]. Despite being a valid subject, and the selectivity of HDAC3 is crucial to minimize off-target toxicity, the effects of certain powerful HDAC3 inhibitors on other HDACs have not been investigated [22]. Since HDAC3 is the primary HDAC enzyme in the NCoR/SMRT complex, additional HDACs can be recruited in a transcription factor-specific or context-specific way. In particular, Class II HDACs (4, 5, 7, and 10) have been known to interact with HDAC3 in the NCoR/SMRT complexes [16].

Therefore, it is important to evaluate not only the potency of inhibitors against HDAC3, but also their selectivity for other HDACs, so that further changes to the relevant lead compounds can be made to produce more effective and highly selective HDAC3 inhibitors for the development of novel therapeutics for different HDAC3-related disorders [22]. The efficacy of HDAC3 selective inhibitors and their specificity in tissues and cells have not been fully investigated, which may limit their clinical application. Another issue is how to predict patients' sensitivity and clinical response towards HDAC3 inhibitors in clinical practice [106]. Therefore, researching these issues to explore the therapeutic potential and effectiveness of HDAC3 will contribute to the development of strategies and drugs for the treatment of atherosclerosis.
